# Exploring Missed Nursing Care in the NICU: Perspectives of NICU Nurses in Saudi Arabia’s Eastern Health Cluster

**DOI:** 10.3390/pediatric15040052

**Published:** 2023-10-02

**Authors:** Nasreen Alsalem, Fatima Abu Rashid, Saleh Aljarudi, Mohammed I. Al Bazroun, Roqayah Mirza Almatrouk, Fatimah M. Alharbi, Lames Al Mansour, Nahid Baker Abuzaid

**Affiliations:** 1 Maternity and Children Hospital, Dammam 32253, Saudi Arabia; faburshaid@moh.gov.sa (F.A.R.); saljarudi@moh.gov.sa (S.A.); ralmatrouk@moh.gov.sa (R.M.A.); falharbi42@moh.gov.sa (F.M.A.); nbabuzaid@moh.gov.sa (N.B.A.); 2 Qatif Health Network, Qatif 32654, Saudi Arabia; malbazroun@moh.gov.sa; 3 Al Jubail General Hospital, Aljubail 35412, Saudi Arabia; lalmansour@moh.gov.sa

**Keywords:** missed nursing care, neonatal intensive care unit (NICU), nursing care omission, nursing workload, patient acuity, work environment, nursing resources, nursing interventions, patient safety, patient outcomes

## Abstract

(1) Background: Missed nursing care, an omission error characterized by delayed or omitted nursing interventions, poses significant risks to patients’ safety and quality of car.; (2) Methods: This is a quantitative cross-sectional study on 151 nurses who work in NICUs in three main networks in the Eastern Health Province, Saudi Arabia: Dammam (*n* = 84), Qatif (*n* = 53), and Jubail (*n* = 14). The study uses a self-reported questionnaire (MISSCARE) and applies the 5-point Likert Scale. Statistical analysis data were analyzed using SPSS version 23.0. (3) Results: The primary reasons for missed care were shortage of nursing staff and unbalanced patient assignments. Missed nursing care negatively affects job satisfaction and was positively correlated with nurses’ intentions to quit their jobs. Inadequate equipment, supplies, and breakdowns in communication between nurses and other healthcare professionals were also significant factors contributing to missed care. (4) Conclusions: Missed nursing care is associated with overwork, nursing shortages, and lower job satisfaction, impacting the quality of care provided in the NICU. Improving working conditions, nurse staffing, and patient assignment planning should be prioritized to address this issue effectively.

## 1. Introduction

Neonatal intensive care unit (NICU) nurses play a crucial role in providing care to critically ill newborns. However, there is growing concern about missed nursing care in the NICU and its potential consequences. Missed nursing care refers to essential care activities that are not completed or delayed, which can have negative impacts on patient outcomes [1]. The primary objective of all healthcare organizations is to guarantee high-quality care and safe practices. Patient safety is defined by the World Health Organization (WHO) as the prevention or mitigation of errors and harm to patients associated with the provision of health care [2]. With several initiatives to measure and reduce errors, the Institution of Medicine encourages healthcare error surveillance and reporting. There are two main categories of errors: errors made by commission due to the wrong action taken and errors made by omission due to actions not taken or missed [3]. Given that nurses comprise most healthcare providers, they play an essential role in maintaining patient safety [4], actively participating in error detection and prevention. In addition, direct patient interaction and interventions are necessary in order for nursing practice to fulfil its holistic value. Nurses are prone to errors due to the complex and intense nature of nursing practice. Additionally, because of time constraints and a lack of manpower and resources, they must frequently consciously prioritize certain tasks over others [5].

In 2022, Kim and Chae reported that missed nursing care is prevalent among NICU nurses in South Korea [6]. For instance, 93.2% of nurses missed providing neonatal developmental care, which was nearly the most frequently missed care, followed by supporting parents emotionally, which has been missed by 88.1% of NICU nurses. Similar to Gathara et al.’s (2019) [7] and Tubbs-Cooley et al.’s (2014) [8] findings, Kim and Chae (2022) also claimed that the involvement of parents in neonatal care and parents’ education were missed by 83.1% of NICU nurses, which was found to be highly associated with decreased pain control levels and delayed discharge preparation [9]. In contrast with Gathara et al.’s (2019) study, Kim and Chae’s (2022) study showed that assessing vital signs, providing feeding at the prescribed intervals, administering oxygen therapy, and adjusting oxygen concentration were among the least frequently missed nursing care tasks. A cross-sectional study conducted by Gathara et al. (2019) on 216 neonates at six hospitals in Nairobi and Kenya concluded that the average completion rate of nursing tasks was 60%, which could adversely impact neonatal safety and outcomes. Examples of missed nursing interventions from the same study included monitoring vital signs, feeding, fluid administration, oxygen therapy, parental education, and poor communication between the parents and nurses and among the health care team, which, consequently, could negatively influence the parent’s experience and the neonate’s early recovery and discharge [7,8,9,10]. Furthermore, nursing care omission was highly associated with medication errors, hospital readmission, pressure ulcers, nosocomial infections, and mortality [10]. Other factors have also been identified as contributing to missed nursing care in the NICU. Tawfik et al., in 2016, found a positive association between perceptions of working too hard and healthcare-associated infections (HAIs) in the NICU [11]. This suggests that burnout and excessive workload can impact the quality of care provided by NICU nurses. In addition, a study conducted by Lake E.T. et al. in 2018 highlighted the role of system factors in contributing to missed nursing care in the NICU; they found that system factors such as personnel staffing and scheduling can affect the ability of nurses to deliver all of the required care [12]. A recent study by Utomo et al. (2022), intended to address the issue of missed nursing care in the NICU, emphasized the need for appropriate interventions in limited resource settings in order to reduce the amount of missed nursing care in the NICU. Therefore, identifying the factors associated with missed nursing care can inform the implementation of interventions to improve care delivery in the NICU [13]. The consequences of missed nursing care in the NICU can have significant implications for both infants and nurses. Infants may experience adverse outcomes, including delayed or inadequate pain management, compromised nutrition, increased risk of infection, and delayed developmental progress [14]. Missed nursing care can also contribute to increased stress and burnout among NICU nurses, affecting their job satisfaction and overall well-being.

Despite the importance of missed nursing care and its predictors in Saudi Arabia, there has been little research on this topic. Few studies have explored the relationship between the practice environment and safety as it relates to missed nursing care. Therefore, this study aims to fill this gap in the literature by explaining the dimensions and related factors of missed nursing care in the neonatal intensive care unit in the Eastern Province of Saudi Arabia.

## 2. Materials and Methods

### 2.1. Study Design

This cross-sectional questionnaire was conducted via an online survey between January 2023 and March 2023 in three main hospitals in the Eastern Province of Saudi Arabia, namely, Dammam, Qatif, and Jubail, using an online Google Form sent to healthcare professionals licensed to work as bedside nurses in the NICU. The study used a non-probability convenience sample. The inclusion criteria were limited to nurses working in the NICU who provide direct patient care, with a minimum experience of six months to two years. Nurses who do not work in the NICU, who do not have a role in direct nursing care in the NICU, who have less than six months of experience, or who are from other specialties were excluded. Ethical approval was obtained from the Maternity and Children’s Hospital Ethical Committee (IRB log number: NR-NICU-2022-001).

Yamane’s sample size formula n = N (1 + N × e^2^) was used to calculate the appropriate sample size, with 95% CI, 5% margin of error, and *n* = 148. A total of 151 participants completed the survey, with a response rate of 151/235.

### 2.2. Data Collection Tool and Validation

The *MISSCARE* questionnaire was adapted for data collection in this study with permission from the original authors—Kalisch B.J. and Williams R. (2009) [15]—and reviewed by three experts before distributing. The items in the first part were divided into six sections, with each one containing several question items (assessment: 8 items; intervention: 13 items; and planning: 3 items) using the five-point Likert scale (never missed, rarely missed, occasionally missed, frequently missed, always missed) and using four degrees of the reason for missed nursing care (with respect to communication (8 items), material resources (3 items), and labour resources (3 items)): not a reason for missed care, minor reason, moderate reason, and significant reason.

A pilot study was conducted with a sample of 16 nurses to confirm the validity of the test using Cronbach’s alpha. The Cronbach’s alpha coefficients for the first and second sections were found to be 0.93 and 0.95, respectively.

### 2.3. Statistical Analysis

Analysis was performed using SPSS version 23. Both descriptive and inferential statistics were carried out. Descriptive statistics include frequency, percentage, and mean and standard deviation (SD). Inferential statistics include Pearson correlation for current job satisfaction and logistic binary regression for the variables that may predict missed nursing care.

## 3. Results

### Sociodemographic Data and Workload

A total of 151 nurses took part in the study, and all participants were female, with 55.6% being between 25 and 34 years old. Of the participants, 84 (55%) were from Dammam and 53 (35%) were from Qatif cities. A total of 115 (76.2%) held a nursing bachelor’s degree, and 66 (43.7%) had more than 10 years of work experience. The vast majority (122, 80%) of the participants worked on a rotating shift, and 91 (60.3%) worked two weekends per month, with 86 (57.0%) taking an average of three days off per week and 61 (40.4%) taking an average of one extra duty shift. Additionally, of the participating nurses, 108 (68.2%) were not given absences in either day work or shift work in the last three months. A total of 63 (41.7%) participants felt that the unit staffing was adequate 75% of the time, and 98 (64.9%), at the time of the questionnaire, had no plan to resign or transfer to another unit (Table 1).

The order below shows the answers for frequently and always missed, respectively. The most frequently missed elements of nursing care, as reported by the participants, were as follows.

Tables S1–S6 present the study participants’ responses, in terms of frequency and percentage, to the different the study dimensions, including assessment (Table S1), intervention (Table S2), planning (Table S3), communication (Table S4), material resources (Table S5), and labor resources (Table S6). For these, please consult the supplementary file.

As illustrated in Table 2, in descending order, the materials, labor, and communication subscales were perceived to be the highest by the study participants; whereas, in descending order, assessment, intervention, and planning scored the lowest as perceived by the study participants in the current study.

The correlation among the sociodemographic and participant work condition variables and missed nursing care was significantly positive for “Age categories” (r = 0.235, *p* < 0.01), “Satisfaction with the extra duty shift” (r = 0.267, *p* < 0.01) and “Intention of leaving current position (resignation, transfer, end of contract, etc.)” (r = 0.41, *p* < 0.01) (Table 3).

Logistic regression was carried out to identify the sociodemographic predictors of missed nursing care (Table S7 (see supplementary file)). Two predictors were found to be statistically significant predictors of missed nursing care: days or shifts missed in the past three months (*p* = 0.04) and intention to leave current position (*p* = 0.0001). The other sociodemographic predictors, including age, health organization, education level, professional experience, current hospital experience, shift, weekend days worked per month, average days off per week, and satisfaction with the load of work in the unit were not found to be statistically significant predictors (*p* > 0.05).

## 4. Discussion

Missed care results when any element of a patient’s care, including clinical, psychological, and administrative nursing care, is skipped (in whole or in part) [15]. Kalisch (2006) defined missed nursing care or rationing nursing care as nursing care that was delayed or omitted [16]. Examples of missed nursing care include, but are not limited to, delayed feeding, breastfeeding counselling [17], parent education, and discharge planning [18]. Missed care is linked to patient safety principles [19]. Therefore, the first step toward improving NICU safety is research on the prevalence of missed care in the NICU. Research on NICU missing care could serve as a diagnostic tool, identifying which NICU behaviors most demand improvement [20] and addressing triggers.

In this study, the most frequently missed care activity related to patient assessment was failure to complete the full documentation of all necessary data. Nurses’ documentation serves diverse purposes that include ensuring continuity of care and effective communication between caregivers, presenting legal evidence of the provided care, and reflecting the quality of patient care. Failure to achieve a high level of accurate and precise nursing documentation can lead to significant professional and patient care consequences. Numerous factors, chief among them being a nurse’s lack of knowledge about the effects of documentation on nursing practice and patient outcomes, can contribute to this failure. Other factors that prompt failure to document include limited nurses’ competence, motivation, and confidence; ineffective nursing procedures; and inadequate nursing auditing, supervision, and staff development [21]. Promoting effective documentation requires the hospital administration to facilitate the process of documentation. This topic requires extensive research in order to determine the individualized causes and then to plan accordingly. One of the most successful action plans discussed in the literature is to offer easy access to an individualized nurses’ workstation, or computers on wheels (COWS), which provides medical professionals and hospital staff with an efficient way to easily document a patient’s status, dispense medication, and manage almost all aspects of patient care on time.

Another frequently missed care activity related to patient assessment was found to be IV site care and assessment, according to hospital policy and intake and output monitoring. Neonates in the NICU are at high risk of extravasation damage, posing varying degrees of morbidity, pain, infection, and deformation and subsequently increasing the length of stay and cost. The best method by which to decrease these complications is through initial prevention. A handful of studies have supported the idea that many of these injuries could be prevented through hourly IV site assessment and prompt removal when evident [22]. Likewise, accurate intake and output monitoring required continuous and high-vigilance monitoring and checking. Missing such basic tasks can be associated with a lack of skilled and well-trained supportive services and personnel, such as healthcare assistants (HCAs). Looking at the number of procedures and interventions to be given to one neonate in the NICU, frontline nurses need enormous support from the unit leaders in undertaking such continuous hourly assessments. This can be achieved by involving the shift leading and clinical educators in the process of the hourly assessment, ensuring the availability of supplies that facilitate the process for the nurses, such as IV site dressing that will not require the removal of any additional bandage over the site which could cause unnecessary distress to the neonate [23]. In addition, nurses’ awareness of the problem can be helpful in ensuring their compliance. Identifying IV extravasation as a problem in the unit, sharing the event of the extravasation with the nurses, discussing the leading cause and complication, and listening to the concerns and struggles that led them to fail the hourly assessment can be helpful in gaining their compliance.

From the intervention perspective, the most frequently missed care activities are individual needs: skin-to-skin contact (kangaroo care, KC), assessing needs according to the new-born individualized developmental care and assessment program (NIDCAP), and mouth care. These findings are consistent with most of the missed care literature, which suggests that these care activities are missed most frequently. While some barriers toward Kangaroo Care and NIDCAP relate to the deficiency of nurses’ information, knowledge, and experience, other barriers include time constraints, safety and security concerns, and nurses’ beliefs and attitudes. Therefore, nurses must have confidence in their own abilities and judgment to take the right action, to understand the benefit of KC, and to accept it [24]. In addition, the implementation of NIDCAP requires significant effort from all professionals involved. The lack of coordination among different professionals is often considered the main obstacle [25]. Moreover, there is a need for additional and ongoing research to describe KC in various countries, especially in the Middle East [26].

In the case of oral care for ventilated babies, it is possible that the shortage of published articles on the neonatal incidences of ventilator-associated pneumonia (VAP) and evidence-based interventions for VAP prevention in NICU settings contributes to a perception among NICU nurses that oral care is not a high-priority nursing care activity. It is also possible that missed oral care is related to a lack of clarity regarding oral care responsibility between bedside nurses and respiratory therapists [27]. Furthermore, lack of organizational support and the absence of clear protocols and standardized oral care techniques are obstacles to maintaining high compliance in oral care among NICU nurses [28].

As for failure to participate in planning, participating in care decisions (multidisciplinary team) whenever held was also identified as a frequently missed care activity in this study. This can be attributed to work overload and a lack of time to participate in such meetings. In addition, nurses will probably have an unclear perception of their role in the process of shared clinical decision-making and their contribution to the therapeutic process [29].

The least common missed care activities were basic care, i.e., checking or changing diapers, dressing or wound care, and bedside glucose monitoring as ordered. The findings of this research are similar to those of previous research on the same topic. Patient care activities with time-sensitive and core tasks need to receive higher priority than needs that may be perceived as time-consuming and not urgent in terms of patient safety, such as comfort care and parental emotional support, and thus there may be a perception that missing them will not have serious impacts on patients’ health [8,9,10,11,12,13,14,15,16,17,18,19,20,21,22,23,24,25,26,27,28,29].

Furthermore, nurses’ reports of missed nursing care reflect an implied prioritization of tasks based on motivations (or disincentives) implemented by hospitals to modify clinician behaviour. For example, there is a continuous audit and follow-up from the infection control department on wound dressing, which might result in the impression among nurses that wound care dressing is an activity of high priority compared to other care activities.

Comparing the most common missed care with the least common missed care reflects an important ethical consideration as the majority of the missed care activities are activities that will involve more developmental and emotional aspects and thus cannot be observed physically compared to the least commonly missed care activities that involve physical changes that can be immediately observed and followed up upon when missed.

The most significant reason for missed care was the inadequate number of staff. Another reason was unbalanced patient assignments. Missed nursing care is associated with overwork and a nursing shortage. Moreover, this study found that approximately 35.1% of participants had planned to leave their current position within six months to a year. Missed nursing care had a significant positive correlation with nurses’ intention to leave their jobs. Additionally, missed nursing care was found to have a negative correlation with job satisfaction.

Staff shortages directly result in increased workload and unbalanced patient assignment for the remaining nurses, who are then likely to be dissatisfied and leave themselves. According to Alotaibi, Saudi nurses would be more satisfied with their jobs if their workload was reduced. Job satisfaction and the quality of the care provided are strongly associated with each other [30].

The overall level of job satisfaction among health care providers in Saudi Arabia was low [31]. Moreover, a higher workload leads to absenteeism. There are a range of cultural factors that contribute to this issue among national nurses [32]. Therefore, identifying the reasons that affect job satisfaction levels may reduce excessive staff turnover, enhance nurses’ work experiences, and boost retention rates [33].

As a result, there is a strong need for a comprehensive plan that focuses on improving the hospital work environment to attract, recruit, and train high-quality nursing staff as per the standard requirements of each department, especially ICUs, and to retain them by increasing their performance and, consequently, their job satisfaction. Healthcare in Saudi Arabia requires leaders to efficiently manage the various issues associated with nursing workforce challenges [32]. Part of such a plan should include a general review of the human resources guidelines in terms of nurse salaries, staffing and scheduling, absenteeism, sick leave penalties, and regulations. NICU nurses’ working environments should be improved in order to ensure adequate time for nursing activities [6].

Missed nursing care due to a staff shortage can be mitigated through improved teamwork. Another solution is to implement a pickup/reliever nurse, in which a nurse can officially, efficiently, and effectively hand off required care (e.g., an oral feeding) to the assigned pick-up nurse to make changes and attend to other high-priority patient situations. Additionally, the implementation of on-call nursing staff may help units adjust to demands and reduce missed care.

Moreover, supplies and equipment not functioning properly and not being available when needed were also significant reasons for missed care. A lack of working equipment has a devastating effect on healthcare. Lack of proper management, availability, and utilization of medical equipment limited the capacity of health institutions to deliver adequate health care. Purchasing devices at cheap prices, a lack of training on how to operate devices, a lack of accountability, power interruptions, staff work overload, a lack of maintenance experts, and an inappropriate referral system were among the reported reasons influencing the availability and utilization of medical devices [34].

The nurse reliever (during break time) did not communicate that care was not provided, and tension or communication breakdowns with the medical staff were also a significant reason for missed care. Effective clinical communication, which results in the timely, accurate, and appropriate transfer of information, is the main aspect of ensuring continuity of care. This reflects the failure of trust and good communication between nurses themselves and between nurses and other healthcare professionals. Mapping the process allows us to identify the gap. Using an effective tool for patient handoff to ensure accountability and the reliability of information will have an impact on the process. Building a culture of responsibility, accountability, and cooperation might require time, but it has a significant impact on improving missed care activities.

The findings of this study suggest that additional personnel and resources are needed in Saudi Arabian NICUs in order to prevent missed nursing care activities. Having enough staff and resources such as equipment and materials can help shorten the time it takes to complete crucial tasks. Effective communication between medical professionals and nurses is crucial for providing all necessary care duties. Improved documentation methods are important for ensuring that all important data are properly recorded, for enhancing continuity of care, and for facilitating the identification of missed nursing care actions. Comprehensive interventions are needed in order to address missing nursing care in the NICU. Strategies to improve the work environment should include appropriate staffing, clear protocols, and standardized approaches for vital activities such as mouth care. Collaboration and relief for nurses or on-call personnel can help reduce missed treatment due to workload and time restrictions. Communication and a culture of responsibility and accountability among healthcare personnel can also aid in reducing missed care. By addressing these issues, NICU nurses may experience higher work satisfaction and be less likely to leave their profession. High nursing staff turnover rates can jeopardize patient care. Therefore, it is important to prioritize these interventions in order to improve the overall quality of care in the NICU. The limitations of this study included the use of a cross-sectional design and self-administered questionnaire. Therefore, further studies with more objective instruments are recommended.

## 5. Conclusions

This is the first study to address missing care activities in the NICUs of governmental hospitals in Saudi Arabia’s Eastern Province. The most often missed activities linked to nursing care evaluation were failure to complete all mandatory data documentation, IV site care assessment, and intake/output monitoring. The most often missed care activities related to the intervention portion of nursing care were individual needs (skin-to-skin contact, kangaroo care) and basic needs (mouth care). Other basic care activities, including diapering or changing, dressing or wound care, and bedside glucose monitoring, were the least frequently skipped. Human resource limitations were the most significant cause of missed care. Unbalanced patient assignments were another reason. Missed nursing care is associated with overwork and nursing shortages, as well as poor job satisfaction and nurses’ intent to leave their positions. Furthermore, materials and equipment that were not working well or were not available when needed were also key factors with respect to missed care. Missed care activities in the NICU were also caused by ineffective communication between nurses and between nurses and healthcare providers.

These findings are significant because they are consistent with the greater body of data on missed care. In managing their work, nurses prioritize safety and core care over developmental care. Missed care is not an indication of negligence or poor-quality nursing care, but rather of nurses’ actions influenced by the unit’s culture and surroundings. Hence, it is recommended that the working conditions and environment for NICU nurses should be changed so that nurses have enough time to complete missed nursing activities. Treatments that improve nurses’ abilities to provide NICU care should be prioritized over educational and awareness treatments, as they may be more beneficial. Such interventions could include attempts to enhance nurse staffing and patient-to-nurse ratios in the NICU, as well as strategies with which to help unit management arrange patient assignments more effectively. Optimizing the recruitment process, allocating resources, and implementing successful nurse retention programs are some strategies that may help to reduce missing nursing care. Future research is required in order to determine the causes and implications of missing nursing care in NICUs.

## Figures and Tables

**Table 1:** Baseline sociodemographic characteristics (*n* = 151).

Characteristics	Frequency	Percent
Age categories		
<25 years	4	2.6
25–34	84	55.6
35–44	48	31.8
45–54	11	7.3
55–64	4	2.6
Health Organization/Working area:		
Qatif	53	35.1
Jubail	14	9.3
Dammam	84	55.6
Highest nursing degree		
Nursing diploma	35	23.8
Bachelor’s degree	115	76.2
MSN with specialization	1	0.7
Professional experience		
6 months–2 years	7	4.6
2–5 years	30	19.9
6–10 years	48	31.8
>10 years	66	43.7
Experience in the current unit		
6 months–2 years	17	11.3
2–5 years	47	31.1
5–10 years	44	29.1
>ten years	43	28.5
Working hours		
Regular shift	29	19.2
Rotating shift	122	80.8
Weekend days worked per month		
1 weekend	25	16.6
2 weekends	91	60.3
3 weekends	30	19.9
4 weekends	5	3.3
Average (days–off) per week		
2 days	56	37.1
3 days	86	57.0
4 days	7	4.6
5 days	2	1.3
Satisfaction with the extra duty shift		
None	69	45.7
1 day	61	40.4
2 days	17	11.3
3 days	3	2.0
5 days	1	0.7
Days or shifts absent in the past three months		
None	103	68.2
1 day	35	23.2
2 days	13	8.6
Satisfaction with the load of work in the unit		
25%	27	17.9
50%	52	34.4
75%	63	41.7
100%	9	6.0
Intention of leaving current position (Resignation-transfer-end of contract …etc.)		
No plan to leave	98	64.9
In the next 6 months	53	35.1

**Table 2:** Descriptive statistics of scale dimensions.

Subscale	Mean ± SD
Assessment	1.73 ± 0.75
Intervention	1.70 ± 0.74
Planning	1.67 ± 0.76
Communication	2.57 ± 0.93
Materials	3.03 ± 1.00
Labor	3.02 ± 1.04

**Table 3:** Correlations among nursing missed care variables.

		1	2	3	4	5	6	7	8
1	Age categories	1.000							
2	Health Organization/Working area	−0.028	1.000						
3	Experience in the current unit	0.408 **	0.027	1.000					
4	Weekend days worked per month	−0.046	0.254 **	−0.107	1.000				
5	Off days per week	0.003	0.164 *	−0.058	−0.039	1.000			
6	Satisfaction with the extra duty shift	0.131	−0.055	0.052	0.039	0.003	1.000		
7	Satisfaction with the load of work in the unit	0.082	−0.060	0.060	−0.038	−0.114	−0.056	1.000	
8	Intention of leaving current position	0.154	0.166 *	0.047	−0.085	0.085	0.124	−0.247 **	1.000
9	Total nursing missed care	0.235 **	0.100	0.082	0.086	0.129	0.267 **	−0.221 **	0.410 **

** Indicates significant at *p* > 0.01; * Indicates significant at *p* > 0.05. 1. Age categories; 2. Health Organization/Working area; 3. Experience in the current unit; 4. Weekend days worked per month; 5. Days off per week; 6. Satisfaction with the extra duty shift; 7. Satisfaction with the load of work in the unit; 8. Intention of leaving current position; 9. Total nursing missed care.

## Data Availability

Data used and analyzed in this study will be promptly available for the publisher upon request.

## Supplementary Materials

The following supporting information can be downloaded at https://www.mdpi.com/article/10.3390/pediatric15040052/s1: Table S1: Assessment; Table S2: Interventions; Table S3: Planning; Table S4: Communication; Table S5: Material Resources; Table S6: Labor Resources; Table S7: Regression (*n*= 151), testing 12 sociodemographic variables in predicting missed nursing care.
