# Supplementary material for: Exploring Missed Nursing Care in the NICU: Perspectives of NICU Nurses in Saudi Arabia’s Eastern Health Cluster
Source: Pediatr Rep. 2023 Oct 2;15(4):571–81. doi: 10.3390/pediatric15040052 (PMC10594518; doi:10.3390/pediatric15040052)
Supplement: Supplementary file 1 [file pediatrrep-15-00052-s001.zip › pediatrrep-2492892-supplementary.pdf]

Supplementary File

Exploring Missed Nursing Care in the NICU: Perspectives of NICU Nurses in Saudi Arabia's Eastern Health Cluster

Table 1S, 2S, 3S, 4S, 5S and 6S presents the study participants responses in frequency and percentage to the different items of the study dimensions including assessment (Table1S), intervention (Table 2S), planning (Table 3S), communication (Table 4S), materials resources (Table 5S) and labor resources (Table 6S).

Table 1S: Assessment

Item	Never missed	Rarely missed	Occasionally missed	Frequently missed	Always missed
Full documentation of all necessary data	42 (27.8%)	65 (43%)	24 (15.9%)	19 (12.6%)	1 (0.7%)
IV site care and assessment according to hospital policy	72 (47.7%)	46 (30.5%)	15 (9.9%)	15 (9.9%)	3 (2%)
Re-calculated intrave0us infusion rate	87 (57.6%)	32 (21.2%)	21 (13.9%)	9 (6%)	2 (1.3%)
Monitoring intake/output	73 (48.3%)	45 (29.8%)	19 (12.6%)	11 (7.3%)	3 (2%)
Vital signs assessed as policy\ patient condition	95 (62.9%)	33 (21.9%)	18 (11.9%)	5 (3.3%)	0
Focused reassessment according to patient condition	82 (54.3%)	41 (27.2%)	22 (14.6%)	5 (3.3%)	1 (0.7%)
Bedside glucose monitoring as ordered	96 (63.6%)	39 (25.8%)	13 (8.6%)	2 (1.3%)	1 (0.7%)
Patient assessments performed each shift	93 (61.6%)	35 (23.2%)	20 (13.2%)	2 (1.3%)	1 (0.7%)

Table 2S: Interventions

Item	Never missed	Rarely missed	Occasionally missed	Frequently missed	Always missed
Assess effectiveness of medications	82 (54.3%)	42 (27.8%)	21 (13.9%)	3 (2%)	3 (2%)
PRN medication orders acted -on within five minutes	75 (49.7%)	50 (33.1%)	18 (11.9%)	6 (4%)	2 (1.3%)
Medications administered within 30 minutes before or after scheduled time	77 (51%)	60 (39.7%)	9 (6%)	2 (1.3%)	3 (2%)

Skin to skin contact (kangaroo care)	78 (51.7%)	21 (13.9%)	30 (19.9%)	7 (4.6%)	15 (9.9%)
Response to sound /light alarm of medical devices	82 (54.3%)	38 (25.2%)	18 (11.9%)	8 (5.3%)	5 (3.3%)
Emotional Support to patient and/or family	87 (57.6%)	32 (21.2%)	21 (13.9%)	10 (6.6%)	1 (0.7%)
Assist mother with breast feeding	88 (58.3%)	33 (21.9%)	16 (10.6%)	12 (7.9%)	2 (1.3%)
Assess needs according to NIDCAP(Newborn Individualized Developmental Care and Assessment Program)	76 (50.3%)	36 (23.8%)	25 (16.6%)	11 (7.3%)	3 (2%)
Incubator care	86 (57%)	37 (24.5%)	17 (11.3%)	6 (4%)	5 (3.3%)
Turning patient every two hours	81 (53.6%)	44 (29.1%)	14 (9.3%)	11 (7.3%)	1 (0.7%)
Mouth Care	77 (51%)	44 (29.1%)	14 (9.3%)	12 (7.9%)	4 (2.6%)
Eye Care	89 (58.9%)	33 (21.9%)	17 (11.3%)	11 (7.3%)	1 (0.7%)
Umbilical care	79 (52.3%)	44 (29.1%)	16 (10.6%)	8 (5.3%)	4 (2.6%)
Warming the milk before feeding the patient	96 (63.6%)	32 (21.2%)	13 (8.6%)	4 (2.6%)	6 (4%)
Patient bathing/skin care	92 (60.9%)	38 (25.2%)	14 (9.3%)	6 (4%)	1 (0.7%)
Dressing /wound care	95 (62.9%)	41 (27.2%)	12 (7.9%)	3 (2%)	0
Check /Change diaper	107 (70.9%)	31 (20.5%)	11 (7.3%)	2 (1.3%)	0

Table 3S: Planning

Item	Never missed	Rarely missed	Occasionally missed	Frequently missed	Always missed
Parents teaching	79 (52.3%)	46 (30.5%)	18 (11.9%)	8 (5.3)	0

Participating in care decision (multidisciplinary team) whenever held	71 (47%)	51 (33.8%)	20 (13.2%)	9 (6%)	0
Ensuring discharge planning	91 (60.3%)	40 (26.5%)	16 (10.6%)	4 (2.6%)	0

Table 4S: Communication

Item	Not a reason for missed care	Minor reason	Moderate reason	Significant
Unbalanced patient assignments	26 (17.2%)	35 (23.2%)	31 (20.5%)	59 (39.1%)
Inadequate hand-off from previous shift	27 (17.9%)	45 (29.8%)	43 (28.5%)	36 (23.8%)
Other departments did not provide the care needed	40 (26.5%)	40 (26.5%)	35 (23.2%)	36 (23.8%)
Lack of back up support from team members	26 (17.2%)	55 (36.4%)	32 (21.2%)	38 (25.2%)
Tension or communication breakdowns with other ancillary/support departments	36 (23.8%)	47 (31.1%)	30 (19.9%)	38 (25.2%)
Tension or communication breakdowns within the nursing team	37 (24.5%)	41 (27.2%)	32 (21.2%)	41 (27.2%)
Tension or communication breakdowns with the medical staff	35 (23.2%)	33 (21.9%)	36 (23.8%)	47 (31.1%)
Nurse reliver (during break time) did not communicate that care was not provided	33 (21.9%)	36 (23.8%)	34 (22.5%)	48 (31.8%)

Table 5S: Material Resources

Item	Not a reason for missed care	Minor reason	Moderate reason	Significant
Medications were not available when needed	20 (13.2%)	43 (28.5%)	30 (19.9%)	58 (38.4%)
Supplies/ equipment not available when needed	15 (9.9%)	33 (21.9%)	22 (14.6%)	81 (53.6%)
Supplies/ equipment not functioning properly	17 (11.3%)	29 (19.2%)	20 (13.2%)	85 (56.3%)

Table 6S: Labor Resources

Item	Not a reason for missed care	Minor reason	Moderate reason	Significant
Inadequate number of staff	18 (11.9%)	23 (15.2%)	26 (17.2%)	84 (55.6%)
Urgent patient situations	24 (15.9%)	30 (19.9%)	24 (15.9%)	73 (48.3%)
Unexpected rise in patient volume and/or acuity on the unit	21 (13.9%)	26 (17.2%)	30 (19.9%)	74 (49%)
Insufficient numbers of Health care assistant and/or ward clerk	27 (17.9%)	29 (19.2%)	20 (13.2%)	75 (49.7%)

Table 7S: Regression (n= 151), testing 12 sociodemographic variables in predicting missed nursing care ,

**Characteristics**	**OR**	**95% CI**	***p-value***
Age			
34 and younger vs. older than 34	1.169	(0.331-4.121)	0.80
Health organization			
Dammam vs Jubail	1.990	(0.457 - 8.658)	0.33
Qatif vs. Jubail	1.208	(0.364 - 4.012)	0.75
Education level			
Diploma vs. bachelor	2.064	(0.746 -5.715)	0.16
Professional experience			
6 years and more vs. 5 years and less	2.490	(0.667 - 9.302)	0.17

Current hospital experience<br>*5 and less vs. 6 and more*	1.444	(0.458 - 4.559)	0.53
Shift<br>*Regular vs. rotation*	1.404	(0.505 – 3.903)	0.51
Weekend days worked per month<br>*Three and f vs. one and two*	1.223	(0.439 - 3.407)	0.70
Avrage days off per week<br>*More two vs. two*	1.132	(0.482 - 2.658)	0.77
Days or shifts absent in the past three months<br>*One and less vs. two and more*	2.299	(1.035 - 5.105)	0.04
Satisfaction with the load of work in the unit<br>*50% and less vs. More than 50*	2.188	(0.941 - 5.089)	0.06
Intention to leave current position<br>*In 6 months vs. no plan*	5.792	(2.495 - 13.445)	0.0001
